# Kidney disease knowledge among patients visiting the nephrology clinic in the Kingdom of Saudi Arabia

**DOI:** 10.1097/MD.0000000000038593

**Published:** 2024-06-14

**Authors:** Sami Alobaidi

**Affiliations:** a Department of Internal Medicine, University of Jeddah, Jeddah, Saudi Arabia.

**Keywords:** chronic kidney disease, knowledge, patients, Saudi Arabia

## Abstract

This study aimed to explore chronic kidney disease (CKD)-related knowledge and its predictors among non-dialysis patients with CKD in the Kingdom of Saudi Arabia (KSA). It was a cross-sectional survey conducted at 2 nephrology centers in KSA. Data were gathered using a survey questionnaire that included sociodemographic information and enquiries about CKD. The questionnaire used to explore CKD knowledge consisted of 24 questions with 3 multiple-choice answers for each question: “True,” “False,” and “I don’t know.” Data were obtained from 185 patients who visited a nephrology clinic. The major study population was drawn from the western region of the Kingdom of Saudi Arabia. The participants’ mean (SD) total renal disease knowledge scores was 12.56 (3.55) out of a maximum of 24 points on the renal disease knowledge assessment. This suggests that the participants, on average, exhibited a moderate level of knowledge regarding renal disease. Most respondents correctly answered questions related to blood and urine tests (90.3% and 89.7%, respectively), living with a single kidney (88.1%), kidney function in blood cleansing (83.8%), risk factors like diabetes (82.7%), and hypertension (80%). Additionally, they recognized symptoms such as water retention (85.9%) and fatigue (61.6%) and the potential of certain medications to slow chronic kidney disease progression (72.4%). However, fewer respondents correctly identified nausea/vomiting (31.4%) and loss of appetite (31.4%) as signs of kidney disease, the role of kidneys in maintaining blood pressure (58.9%) and bone health (16.2%), and obesity as a risk factor (54.1%). Furthermore, there were notable differences in knowledge scores between genders, with men scoring significantly higher than women (2.05, *P* = .041). In general, the understanding of CKD within the CKD patient community in the KSA was at a moderate level. However, male respondents had a greater understanding of CKD than did female respondents. The findings of this study indicate an urgent need to conduct educational activities to improve CKD knowledge among patients with CKD in the KSA.

## 1. Introduction

Chronic kidney disease (CKD) has emerged as one of the most prominent global health problems in terms of morbidity and mortality globally.^[1]^ The incidence of CKD is increasing globally due to many factors, most importantly the rise in risk factors such as diabetes mellitus, obesity, and hypertension.^[2]^ Studies examining the global prevalence of CKD have shown that approximately 843.6 million individuals are currently affected by CKD stages 1 to 5 worldwide currently.^[3]^ Owing to the high prevalence of CKD risk factors, the incidence of CKD is significantly increasing in the Kingdom of Saudi Arabia (KSA).^[4]^ Recent reports have shown that around 2.4 million people suffer from CKD in the KSA, of which 0.6% to 7.8% are at 3rd stage of CKD.^[2]^ Another recent study also showed that approximately 4.4% of the KSA population suffers from CKD stages 3 to 5.^[5]^ This significant increase in the progression of CKD to end-stage kidney disease (ESKD) in the KSA is likely to persist in the absence of proactive measures to improve knowledge and awareness of CKD in patients.^[1]^ Considering the significant morbidity and mortality associated with ESKD and the enormous economic burden on patients, there is an urgent need to address this public health problem in KSA.^[1,2]^

One of the main goals of CKD treatment is to mitigate disease progression to ESKD to improve longevity and avoid the need for renal replacement therapy or transplantation.^[6]^ CKD progression can be effectively achieved through self-management strategies, such as medication adherence, discontinuation of tobacco products, physical activity, diet control, hypertension (HTN) and diabetes mellitus (DM) control, and avoidance of nephrotoxic drugs.^[1,2]^ Studies have shown that the knowledge level of CKD among patients can significantly influence behavioral modification in favor of effective mitigation of disease progression.^[7,8]^ Furthermore, higher knowledge about the disease condition can also empower patients to make crucial treatment decisions during clinical encounters with their physicians and help them cultivate effective coping strategies to respond to future challenges associated with their illness.^[9]^ Hence, there is a need to systematically study the level of CKD knowledge among patients in various countries to understand the knowledge gaps so that effective strategies can be implemented to address them.

Although studies have shown significantly higher awareness of CKD among patients under nephrology care compared to the general population, knowledge about the disease and its management among patients with CKD has been found to be inadequate.^[10,11]^ Only a few studies have explored knowledge of CKD among patients with CKD in Arab countries. To the best of our knowledge, only one study to date has explored CKD-related knowledge among patients with CKD in KSA.^[2]^ Hence, this study aimed to explore CKD-related knowledge and its predictors among non-dialysis patients with CKD in the KSA. The results of this study can inform knowledge gaps among patients with CKD, which can be utilized for clinical and policy decision making.

## 2. Methodology

The current study was a cross-sectional face-to-face survey conducted at 2 nephrology centers in KSA between October 11, 2022 and February 22, 2023. A purposive sampling technique was used to recruit participants. The institutional ethics committee approved the study protocol and informed consent was obtained from each participant before data collection. The inclusion criteria encompassed all patients attending the nephrology clinic who were willing to participate in the study. Conversely, patients with severe medical conditions that impeded their ability to partake in the study, as well as those who declined to provide consent, were excluded from participation. The sample size was determined using data from a prior study done in KSA investigating kidney disease knowledge among CKD patients.^[2]^

Data were gathered using a survey questionnaire that included sociodemographic information and enquiries about CKD. The questionnaire used to explore CKD knowledge contain 24 questions. There were 3 multiple-choice answers for each question: “True,” “False,” and “I don’t know.” The correct responses received a score of 1, whereas erroneous responses received a score of 0. The option “I don’t know” was considered as lack of knowledge and given a score of 0. The Gheewala et al (2018) designed and validated this questionnaire, which has strong internal consistency and a Cronbach alpha of 0.88 (95% CI: 0.86–0.91).^[12]^ This study was conducted in accordance with the principles of the Declaration of Helsinki.

### 2.1. Statistical analysis

The Statistical Package for Social Sciences (SPSS Inc., Chicago, IL, version 26.0) software was used to analyze the data. The sociodemographic and clinical variables of the participants were described using descriptive statistics. Independent sample t-tests and one-way ANOVA were used to analyze the relationship between sociodemographic variables and CKD knowledge scores. Statistical significance was defined as a *P*-value of <.05.

## 3. Results

We collected data from 185 patients who visited the nephrology clinics. The average age of the study participants was 58.17 (±13.58), with ages ranging from 19 to 86 years old. Of the study participants, 71.4% were male, 53% were graduates, and 81.6% were married. Among the study participants, 47.6% had stage 3 CKD. Type 2 DM, HTN, and heart disease accounted for 59.5%, 80.5%, and 15.7% of patients, respectively. Of the study participants, 24.5% were smokers and only 22.7% reported physical activity for >150 min/week. Sociodemographic and clinical variables are summarized in Table 1.

**Table 1:** Participant characteristics.

Characteristics	Participants	
	N	%
Total	185	100
Gender		
Female	53	28.6
Male	132	71.4
Education		
High school or less	51	27.6
Bachelor	98	53
Masters or PhD	36	19.5
Marital status		
Single	8	4.3
Married	81.6	81.6
Divorced/separated/widowed	26	14.1
Chronic kidney disease		
Stage 1 &2	52	28.1
Stage 3	88	47.6
Stage 4	20	10.8
Stage 5	1	0.5
No CKD	24	13
Presence of diabetes		
Type 2	110	59.5
Type 1	4	2.2
No	71	38.4
Presence of HTN		
Yes	149	80.5
No	36	19.5
Presence of heart disease		
Yes	29	15.7
No	156	84.3
Presence of stroke		
Yes	4	2.2
No	181	97.8
Smoker		
Yes	45	24.3
No	108	58.4
Ex-smoker	32	17.3
Physical activity		
None	92	49.7
<150 minutes/week	51	27.6
>150 minutes/week	42	22.7

HTN = hypertension.

The mean (standard deviation) total scores for participants’ knowledge of renal diseases ranged from 3 to 21, with a mean score of 12.56 (3.55). Most participants provided correct answers to various questions: blood tests (90.3%) and urine tests (89.7%) are commonly employed to assess kidney health, individuals can live normally with a single kidney (88.1%), kidneys perform blood purification (83.8%), diabetes is a risk factor for kidney disease (82.7%), hypertension poses a risk for kidney disease (80%), symptoms of kidney disease include water retention (85.9%) and increased fatigue (61.6%), and certain medications can mitigate the progression of chronic kidney disease (72.4%).

However, only a minority of respondents provided accurate responses to certain inquiries: nausea/vomiting (31.4%) and loss of appetite (31.4%) were recognized as signs of kidney disease, the kidneys’ role in maintaining blood pressure (58.9%) and bone health (16.2%) was acknowledged, and obesity was identified as a risk factor for kidney disease (54.1%).

Conversely, the majority of respondents provided incorrect answers to other questions: kidneys’ involvement in maintaining blood sugar levels (79.5%) and protein breakdown (88.6%), being female (78.9%) or having heart disease (67.6%) or experiencing excess stress (78.4%) as kidney disease risk factors, and fever being indicative of kidney disease (82.7%). The distribution of correct responses to each question in the kidney disease knowledge questionnaire is detailed in Table 2.

**Table 2:** Percentage of correct response to individual items on the questionnaire.

Question	Correct response (%)
A person can lead a normal life with one healthy kidney.	88.1
Herbal supplements can be effective in treating chronic kidney disease.	43.2
Certain medications can help to slow-down the worsening of chronic kidney disease.	72.4
What functions do the kidneys perform in the body?	
The kidneys make urine.	53
The kidneys clean blood.	83.8
The kidneys help to keep blood sugar level normal.	20.5
The kidneys help to maintain blood pressure.	58.9
The kidneys help to breakdown protein in the body.	11.4
The kidneys help to keep the bones healthy.	16.2
Which of the following are commonly used to determine health of the kidneys?	
A blood test.	90.3
A urine test.	89.7
A fecal (poo) test.	53.5
Blood pressure monitoring.	55.1
What are the risk factors for chronic kidney disease?	
Diabetes.	82.7
Being female.	21.1
High blood pressure.	80
Heart problems such as heart failure or heart attack.	32.4
Excess stress.	21.6
Obesity.	54.1
What are the signs and symptoms that a person might have if they have advanced chronic kidney disease or kidney failure?	
Water retention. (excess water in the body)	85.9
Fever.	17.3
Nausea/vomiting.	31.4
Loss of appetite.	31.4
Increased fatigue (tiredness).	61.6

According to the results of the bivariate analysis, conducted using independent sample *t*-tests, significant variations were observed in the mean kidney disease knowledge total score between genders. Men scored notably higher than women (2.05, 183, 0.041). Table 3 summarizes the independent *t*-test results. There was no significant mean difference in the total kidney disease knowledge score across age, educational level, marital status, and frequency of physical activity in the one-way ANOVA test.

**Table 3:** Results of the bivariate analysis performed using independent *t*-test between individual participant characteristic and total score.

	Total score mean (SD)	t	Df	*P*-value
Gender		2.05	183	.041
Male	12.89 (3.51)			
Female	11.72 (3.54)			
Hypertension		‐0.154	183	.878
Yes	12.53 (3.66)			
No	12.64 (3.12)			
Heart disease		0.446	183	.656
Yes	12.83 (4.35)			
No	12.51 (4.71)			
Stroke		0.820	183	.413
Yes	14 (3.83)			
No	12.52 (3.55)			

## 4. Discussion

This study was conducted among the CKD patient population in the KSA to understand their level of knowledge regarding CKD, and showed a significant knowledge gap regarding various aspects of CKD in the studied population. However, there is a major knowledge gap regarding kidney function. Only 53% and 16.2% of the study population correctly identified the function of the kidneys in creating urine and maintaining bone health, respectively. A recent study among the general population of the KSA regarding CKD knowledge reported relatively better results; 59.4% and 28.9% of the study population correctly identified the function of the kidney in making urine and maintaining bone health, respectively.^[4]^ More than 70% of the study population incorrectly attributed the kidneys to blood sugar maintenance and protein metabolism. Similar misconceptions regarding the role of the kidney in the control of blood glucose levels were also reported in a recent study of a CKD patient population in the KSA.^[2]^ The respondents, however, had a fair understanding of how the kidneys clear blood and control blood pressure.

Screening for CKD and regular monitoring of kidney health are important public health approaches to prevent CKD progression to more severe stages. Patients’ understanding of the commonly used tests to determine kidney health is critically important in planning and implementing public health interventions to address the growing public health challenge of the increased incidence of end-stage kidney disease. Over 89% of the participants in the study correctly recognized blood and urine tests as methods for evaluating kidney health, while nearly 55% accurately identified blood pressure monitoring. This indicates a substantial level of comprehension. The percentage of correct identification of tests for assessing kidney health was notably higher compared to similar previous studies conducted among the general population in the KSA.^[4,13]^ Similarly, over 80% of participants recognized DM and HTN as risk factors for chronic kidney disease (CKD). However, only 54.1% of the participants correctly identified obesity as a risk factor for CKD, and the majority incorrectly identified stress and female sex as CKD risk factors. These findings are similar to those of previous studies conducted in the general population of KSA.^[4]^ Another finding from this study was that 88.1% of those polled were aware that just one kidney is needed for the maintenance of a normal life, which is a marginal improvement over the general population of KSA.^[4]^

In our study, 72.4% of the study participants knew that medications could help slow down the worsening of CKD, which is an encouraging finding. Yet, 43.2% of the participants in the study held the belief that herbal supplements might be efficacious in managing CKD, highlighting incorrect beliefs among the studied population regarding evidence-based effective treatment of CKD. These findings are much better than those of similar studies on the general Saudi population. A recent investigation into CKD understanding among the general populace of KSA indicated that around 66.9% of respondents held the view that herbal supplements might have efficacy in addressing CKD.^[4]^ Moreover, these findings are much better than those of previously published studies on CKD. A recent study from Canada among patients with advanced CKD reported that more than 60% perceived themselves to know little or nothing about medications that help the kidney.^[14]^ Another study from Nigeria reported that only 65% of study participants correctly identified the role of medications in preserving kidney health.^[1]^ Our results underscore the necessity for effective educational interventions for CKD patients concerning evidence-supported treatment choices, given that a notable portion of participants held misconceptions regarding the efficacy of herbal supplements in CKD treatment.

Earlier research has demonstrated a notable correlation between age, marital status, educational achievement, annual household income, comorbidities, and CKD knowledge. Nonetheless, this study did not reproduce these findings and indicated notably higher CKD knowledge scores among male participants with CKD. Similarly, a recent investigation from Ethiopia examining knowledge about chronic kidney disease among care providers also observed significantly higher knowledge scores among males compared to females.^[15]^

The participants in this study demonstrated poor knowledge of CKD symptoms. Only approximately 30% of the study participants correctly identified nausea/vomiting and loss of appetite as symptoms of CKD. Moreover, only 17.3% of the patients correctly identified fever as not a symptom of CKD. However, 85.9% and 61.6% of the respondents correctly identified water retention and fatigue, respectively, as symptoms of CKD. A previous study of the CKD population in the KSA reported relatively better knowledge regarding the signs and symptoms of CKD.^[2]^ The results of this study suggest that patients’ symptom experiences can influence their knowledge of their CKD symptoms.

The current study had several limitations. As this was a cross-sectional study conducted at only 2 centers, located in the western region, the results cannot be generalized to the CKD population at the national level. A larger multi-site study in a representative population of patients with CKD should be conducted to validate our findings. Another important limitation in our study was nonresponse bias. As this was a face-to-face study conducted in 2 nephrology clinics, we aimed to minimize nonresponse bias by actively engaging with patients during their clinic visits. Despite our efforts, we recognize that nonresponse bias may still exist to some extent.

In summary, the current study used a validated questionnaire to examine CKD knowledge among patients with CKD living in the KSA. Overall, knowledge of CKD among the CKD patient population in the KSA was poor. However, male respondents had a greater understanding of CKD than did female respondents. The findings of this study indicate an urgent need to conduct educational activities to improve CKD knowledge among patients with CKD in the KSA.

## Author contributions

**Conceptualization:** Sami Alobaidi.

**Data curation:** Sami Alobaidi.

**Formal analysis:** Sami Alobaidi.

**Funding acquisition:** Sami Alobaidi.

**Investigation:** Sami Alobaidi.

**Methodology:** Sami Alobaidi.

**Project administration:** Sami Alobaidi.

**Resources:** Sami Alobaidi.

**Software:** Sami Alobaidi.

**Supervision:** Sami Alobaidi.

**Validation:** Sami Alobaidi.

**Visualization:** Sami Alobaidi.

**Writing – original draft:** Sami Alobaidi.

**Writing – review & editing:** Sami Alobaidi.

## References

[1] Okoro RN Ummate I Ohieku JD Yakubu S Adibe MO Okonta MJ. Kidney disease knowledge and its determinants among patients with chronic kidney disease. J Patient Exp. 2020;7:1303–9. 33457579 10.1177/2374373520967800 PMC7786774

[2] Almutary HH. Assessment of kidney disease knowledge among chronic kidney disease patients in the Kingdom of Saudi Arabia. J Ren Care. 2021;47:96–102. 33625797 10.1111/jorc.12363

[3] Kovesdy CP. Epidemiology of chronic kidney disease: an update 2022. Kidney Int Suppl (2011). 2022;12:7–11. 35529086 10.1016/j.kisu.2021.11.003 PMC9073222

[4] Alobaidi S. Knowledge of chronic kidney disease among the population of Saudi Arabia evaluated using a validated questionnaire: a cross-sectional study. Patient Prefer Adherence. 2021;15:1281–8. 34163145 10.2147/PPA.S315369 PMC8214335

[5] Mousa D Alharbi A Helal I. Prevalence and associated factors of chronic kidney disease among relatives of hemodialysis patients in Saudi Arabia. Kidney Int Rep. 2021;6:817–20. 33732996 10.1016/j.ekir.2020.12.029 PMC7938070

[6] Chen TK Knicely DH Grams ME. Chronic kidney disease diagnosis and management: a review. JAMA. 2019;322:1294–304. 31573641 10.1001/jama.2019.14745 PMC7015670

[7] Cavanaugh KL. Prioritizing patient-centered care implementation and research for patients with kidney disease. Semin Dial. 2015;28:131–40. 25470535 10.1111/sdi.12326

[8] Kumela Goro K Desalegn Wolide A Kerga Dibaba F. Patient awareness, prevalence, and risk factors of chronic kidney disease among diabetes mellitus and hypertensive patients at Jimma University Medical Center, Ethiopia. Biomed Res Int. 2019;2019:2383508. 31214611 10.1155/2019/2383508 PMC6535886

[9] Kalantar-Zadeh K Lockwood MB Rhee CM. Patient-centred approaches for the management of unpleasant kidney disease symptoms. Nat Rev Nephrol. 2022;18:185–98. 34980890 10.1038/s41581-021-00518-z

[10] Chu CD Chen MH McCulloch CE. Patient awareness of CKD: a systematic review and meta-analysis of patient-oriented questions and Study Setting. Kidney Med. 2021;3:576–85.e1. 34401725 10.1016/j.xkme.2021.03.014 PMC8350814

[11] Enworom CD Tabi M. Evaluation of kidney disease education on clinical outcomes and knowledge of self-management behaviors of patients with chronic kidney disease. Nephrol Nurs J. 2015;42:363–72; quiz 373. 26462309

[12] Gheewala PA Peterson GM Zaidi STR Jose MD Castelino RL. Public knowledge of chronic kidney disease evaluated using a validated questionnaire: a cross-sectional study. BMC Public Health. 2018;18:371. 29554891 10.1186/s12889-018-5301-4 PMC5859642

[13] Madani BM Halawani M Qashqary M Eid M. Determinants of knowledge about chronic kidney disease at an academic setting in Jeddah: a cross-sectional analytic study. IJMDC. 2022;6:950–6.

[14] Molnar AO Akbari A Brimble KS. Perceived and objective kidney disease knowledge in patients with advanced CKD followed in a multidisciplinary CKD clinic. Can J Kidney Health Dis. 2020;7:2054358120903156. 32110417 10.1177/2054358120903156 PMC7016305

[15] Wolide AD Kumela K Kerga F. Knowledge, attitude, and practices toward chronic kidney disease among care providers in Jimma town:cross-sectional study. BMC Public Health. 2020;20:1079. 32646400 10.1186/s12889-020-09192-5 PMC7346627

